# Effects of repeated low-level red light therapy on myopia progression in children: a systematic review and meta-analysis

**DOI:** 10.3389/fmed.2025.1640403

**Published:** 2025-08-13

**Authors:** Haobo Fan, Jia Yu, Aiming Jiang, Qiumei Wei, Xuemin Zhang, Airui Xie, Junguo Duan

**Affiliations:** ^1^Eye College of Chengdu University of Traditional Chinese Medicine, Chengdu, China; ^2^Department of Optometry and Pediatric Ophthalmology, Ineye Hospital of Chengdu University of Traditional Chinese Medicine, Chengdu, China; ^3^Key Laboratory of Sichuan Province Ophthalmopathy Prevention & Cure and Visual Function Protection with TCM Laboratory, Chengdu, China; ^4^Retinal Image Technology and Chronic Vascular Disease Prevention & Control and Collaborative Innovation Center, Chengdu, China

**Keywords:** systematic review, meta-analysis, myopia, repeated low-level red light, children

## Abstract

**Purpose:**

This study aimed to evaluate the effects of repeated low-level red light (RLRL) therapy in intervening in the progression of myopia in children.

**Methods:**

We searched PubMed, the Cochrane Library, Embase, Web of Science, and CNKI databases for relevant studies published from the inception of the databases to 30 April 2025. Subsequently, studies were screened according to the inclusion and exclusion criteria, and basic information and outcome data of the included studies were recorded. The risk of bias in randomized controlled trials (RCTs) and cohort studies was assessed using the RoB 2.0 tool and the NOS, respectively. Finally, meta-analysis was performed using RevMan 5.4, and meta-regression, sensitivity analysis, and publication bias assessment were conducted using STATA 17.

**Results:**

A total of 20 studies were included in this study, involving 2,638 Chinese children, aged from 3 to 16 years, with a baseline spherical equivalent refraction (SER) ranging between +0.75 and −10.00 diopters. A meta-analysis showed that, compared with the control group, the RLRL group had a slower axial elongation, a lower progression of SER, and a greater increase in subfoveal choroidal thickness (SFCT). The changes in axial length (AL) at the 6th, 12th, and 24th months of follow-up in the RLRL group relative to the control group were −0.22 mm (95% *CI*: −0.25, −0.18; *p* < 0.00001), −0.30 mm (95% *CI*: −0.36, −0.24; *p* < 0.00001), and −0.61 mm (95% *CI*: −0.71, −0.52; *p* < 0.00001), respectively. The corresponding changes in SER at these time points were 0.40 D (95% *CI*: 0.31, 0.50; *p* < 0.00001), 0.61 D (95% *CI*: 0.47, 0.76; *p* < 0.00001), and 1.33 D (95% *CI*: 0.62, 2.03; *p* = 0.0002). Additionally, the changes in SFCT at the 6th and 12th months of follow-up were 31.21 μm (95% *CI*: 22.03, 40.38; *p* < 0.00001) and 29.72 μm (95% *CI*: 19.53, 39.92; *p* < 0.00001), respectively. Meta-regression and subgroup analysis revealed that the baseline SER and treatment frequency primarily contributed to the heterogeneity observed in this study.

**Conclusion:**

This meta-analysis confirmed that RLRL therapy can effectively delay the progression of myopia in children during a 6–24 months follow-up, and the efficacy appears to be directly related to the degree of the baseline myopia and the LRLR treatment frequency. However, a causal relationship has been suggested between retinal damage and LRLR treatments, which requires further investigations.

**Systematic review registration:**

https://www.crd.york.ac.uk/PROSPERO/, identifier CRD420251018947.

## 1 Introduction

Myopia has become a significant public health issue globally, with its incidence rising each year (1, 2). Epidemiological studies indicate that the high rates of myopia not only increase the social and economic burden but also present a considerable challenge to the public health system (3). From a pathological perspective, myopia is primarily characterized by abnormal axial elongation (1). Clinical evidence demonstrates that this pathological change can substantially elevate patients’ risk of developing severe vision-threatening conditions, such as myopic macular degeneration, retinal detachment, cataracts, and open-angle glaucoma (4). It is essential to note that the age of onset for myopia is closely associated with the risk of developing high myopia. For each year that the onset age of myopia in children is delayed, the likelihood of developing high myopia in adulthood is significantly reduced (5). This underscores the importance of early prevention and control of myopia.

Multiple studies have shown that factors such as the duration of near-work activities, work intensity, and light intensity are primary risk factors influencing the occurrence and progression of myopia in children (6). An epidemiological survey conducted during the COVID-19 pandemic revealed that the extended home-stay time and the increased use of electronic screens resulted in a 1.4- to 3-fold increase in the incidence of myopia among children aged 6–8 years (7). Long-term studies have confirmed that a lack of outdoor activities is a significant risk factor for the occurrence and development of myopia in children (8). Increasing the time spent on outdoor activities can effectively prevent or delay the onset of myopia (9–11). Even children who have been engaged in close work for a long time can have a positive impact on myopia prevention and control through intermittent high-intensity outdoor light exposure (12). Lingham et al. (13) suggested that the protective effect of outdoor light on myopia may be related to light intensity and spectral composition. Animal experiments have shown that, compared with other wavelengths of visible light, red light helps reduce the elongation of the vitreous cavity and increase choroidal thickness in rhesus monkeys, thereby delaying their emmetropization process (14).

Low-level laser therapy is a form of phototherapy that applies low-dose red and near-infrared light to induce various molecular, cellular, and tissue effects (15, 16). Researchers have proposed using repeated low-level red light (RLRL) devices to repeatedly direct light onto the retina over a short period to slow the development of myopia in children (17–19). RLRL therapy employs a phototherapy device emitting weak red laser light with a wavelength of 635 nm or 650 nm, an illuminance of 700 or 1,600 Lux, and a laser safety classification of Class 1 or Class 2 (20–22). During treatment, children are required to gaze at a fixed observation port in the instrument, focusing on a stationary red light spot within the irradiation device. The standard treatment regimen consists of two sessions per day (each lasting 3 min), with an interval of at least 4 h between sessions and a minimum frequency of 5 days per week. This treatment method has been validated in multiple previous studies, which have demonstrated that axial length (AL), spherical equivalent refraction (SER), and subfoveal choroidal thickness (SFCT) are all potential indicators of its efficacy in treating myopia in children (19, 23–25). Relevant meta-analyses have demonstrated that RLRL therapy helps slow down the elongation of the eye axis and the increase in refractive power in children with myopia (26–28). However, existing meta-analyses have the following limitations: an insufficient number of included studies, unclear sources of heterogeneity, and potential publication bias, which weaken the statistical efficacy of the study. This study aims to provide more robust meta-analysis results by including the latest RLRL-related studies. Additionally, by analyzing the sources of heterogeneity in results and evaluating the influence of publication bias, the clinical efficacy of RLRL therapy for children’s myopia intervention is systematically evaluated, providing more comprehensive evidence-based medical information for the management of children’s myopia.

## 2 Methods

This study followed the guidelines of the Cochrane Handbook and the Preferred Reporting Items for Systematic Reviews and Meta-Analyses (PRISMA) (29, 30). This study was registered in the International Prospective Register of Systematic Reviews (PROSPERO; ID: CRD420251018947). The PRISMA 2020 checklist is provided in Supplementary File 1.

### 2.1 Search strategy

Two researchers (HF and JY) systematically searched PubMed, the Cochrane Library, Embase, Web of Science, and CNKI databases to collect relevant studies on RLRL therapy for pediatric myopia control. The search covered the period from the inception of the databases until 30 April 2025 and included studies in both English and Chinese. The search formula was (Myopia OR Myopias OR Nearsightedness OR Nearsightednesses) AND (Red light) OR (Low-power laser therapy) OR (Photonic stimulation) OR (Photobiomodulation OR Phototherapy) AND (Child OR Children). These terms were adapted for use in different databases and websites (see Supplementary material for details of the search terms). In addition to the identified studies and relevant systematic reviews, additional studies were included by screening the reference lists of relevant studies and systematic reviews.

### 2.2 Study selection

This study was guided by the PICOS framework: (P) Population: Children with myopia or pre-myopia (31). (I) Intervention/Exposure: The treatment group received RLRL treatment. (C) Comparison: The control group was not treated with RLRL. (O) Outcomes: Between-group differences in changes in outcomes with at least one of the following follow-up periods of more than 6 months: (1) AL (mm); (2) SER (D); (3) SFCT (μm). (S) Study design: randomized controlled trials (RCTs) or cohort studies published as full-length articles in English or Chinese.

Among RCTs and cohort studies, we excluded studies that (1) enrolled adult populations, (2) applied RLRL in combination with other myopia control interventions, or (3) did not report key outcome measures in cases where studies had overlapping patient cohorts; only the study with the largest sample size was retained for the meta-analysis.

### 2.3 Filtrate the articles

Two researchers (HF and JY) independently performed the literature search using the predefined retrieval strategy and compiled their respective findings. Initial screening involved excluding duplicates, case reports, review articles, and other irrelevant publications based on title evaluation to ensure alignment with the study objectives. The inclusion and exclusion criteria were rigorously applied, followed by a comprehensive assessment of full-text articles to determine eligibility for retention. The final selection of studies was cross-verified by the two researchers (HF and JY), and any substantial heterogeneity among the included studies was resolved through consultation with a third researcher (JD) for adjudication of study inclusion.

### 2.4 Data extraction

Essential study characteristics were extracted from all eligible publications, including the authors’ names, study location (country or region), publication year, study design, participant age range, sample size, intervention protocols, study duration, and measured outcomes, to ensure comprehensive data collection for subsequent analysis. This study systematically extracted baseline and endpoint measurements of AL, SER, and SFCT to assess RLRL-induced differences. All extracted data were documented in a standardized Excel spreadsheet (Microsoft Corporation) to ensure systematic data management and facilitate subsequent statistical analysis. When available, pre-calculated change values were directly obtained from original studies. All continuous variables were reported as mean ± standard deviation (*SD*); when presented as interquartile ranges or 95% confidence intervals, appropriate conversions were performed (32). For longitudinal studies, only data from the most extended follow-up period were included to ensure temporal consistency. In the crossover trial, we exclusively analyzed first-phase data preceding intervention switching to preserve methodological rigor and minimize potential carryover effects. Furthermore, duplicate data from overlapping cohorts due to redundant publications in the included studies were appropriately excluded. Data extraction was independently performed by two researchers (HF and JY), and any discrepancies were resolved through discussion until consensus was reached or by consulting a third researcher (JD).

### 2.5 Risk of bias assessment

A comprehensive evaluation of methodological quality was conducted using validated assessment tools appropriate for each study design. For RCTs, the Cochrane Risk of Bias tool (RoB 2.0) was employed to systematically assess randomization processes, allocation concealment, blinding procedures, outcome reporting completeness, and other potential sources of bias. Cohort studies were evaluated using the Newcastle-Ottawa Scale (NOS), with particular attention to participant selection criteria, group comparability, and ascertainment of outcomes and exposures. Based on established quality thresholds, cohort studies achieving an NOS score of 7 or higher were considered methodologically sound. Two researchers (HF and JY) independently evaluated the quality of the included studies, and any disagreements were resolved through discussion with a third researcher (JD) to reach a consensus.

### 2.6 Statistical analysis

The meta-analysis was conducted using Review Manager 5.4 software (Cochrane Collaboration). Mean, *SD*, mean differences (*MD*), and 95% confidence intervals (*CI*) were calculated and used as effect measures for changes in AL, SER, and SFCT. Heterogeneity was assessed using the chi-square test based on *Q* and *I*^2^ statistics. If no significant heterogeneity was observed (*p* > 0.10, *I*^2^ < 50%), a fixed-effects model was applied; otherwise, a random-effects model was used.

Meta-regression, sensitivity analysis, and publication bias assessment were performed using STATA 17.0 (StataCorp, College Station). Meta-regression was employed to explore potential sources of heterogeneity in the meta-analysis results, and subgroup analysis was conducted based on the primary sources of heterogeneity identified. Sensitivity analysis was conducted using the leave-one-out method to investigate heterogeneity further and evaluate the robustness of the pooled estimates. The results were considered robust if the overall effect size remained statistically unchanged upon sequential exclusion of individual studies.

Publication bias was assessed using funnel plots and Egger’s test. The non-parametric trim-and-fill method was applied for studies exhibiting significant publication bias to estimate its potential impact on the meta-analysis outcomes. All results in this analysis were considered significant only with a two-tailed *p*-value of <0.05.

## 3 Results

### 3.1 Literature screening

**Figure 1:**
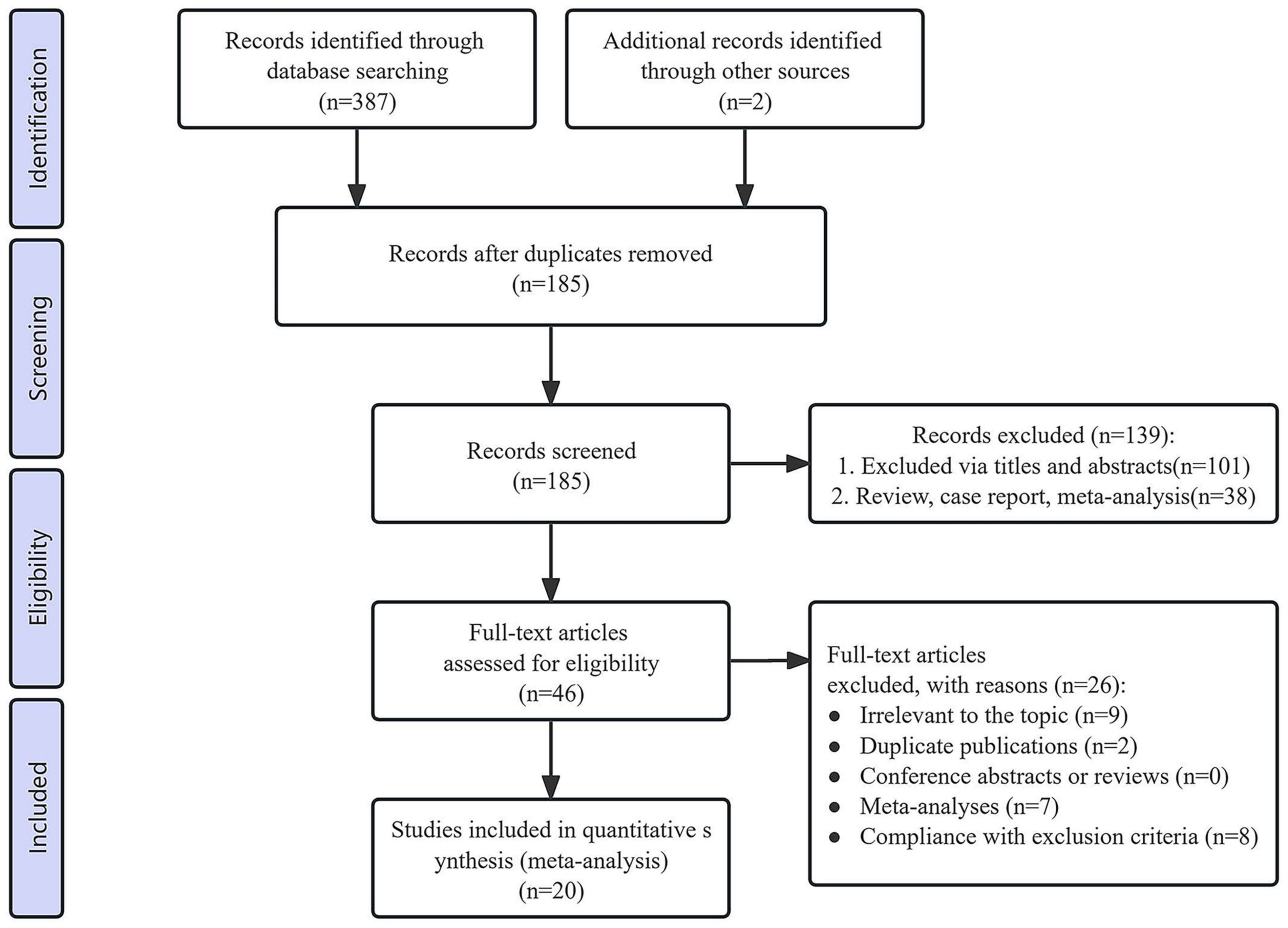
PRISMA flow diagram of the study selection process.

During the initial screening process, a total of 389 studies were identified. After removing duplicates, 185 records remained eligible for screening. Following the title and abstract review, 139 irrelevant records were excluded. Upon full-text evaluation of the remaining 46 records, 26 studies were further excluded due to failure to meet the inclusion criteria. Ultimately, 20 studies [15 RCTs (21, 22, 24, 25, 33–43) and 5 cohort studies (17, 20, 44–46)] were included in the meta-analysis. The PRISMA flow diagram illustrates the study selection process (Figure 1).

### 3.2 Basic characteristics of the included studies and risk of bias assessment

The meta-analysis included 20 studies (15 RCTs and 5 cohort studies), all of which were conducted in China with the following regional distribution: North China (*n* = 9), South China (*n* = 5), East China (*n* = 4), and Central China (*n* = 2). These studies were published between 2021 and 2025, and a total of 2,638 children were enrolled, including 1,405 children in the RLRL group and 1,233 children in the control group, aged from 3 to 16 years, with a baseline SER ranging between +0.75 and −10.00 diopters (Table 1).

**Table 1:** Characteristics of the studies included in the meta-analysis.

Study (author, year)	Countries and regions	Study design	Intervention protocols of RLRL therapy						Sample size	Interventions	Baseline				Follow-up duration	Outcomes
			RLRL therapy device	Laser safety classes	Wavelength (nm)	Illuminance (lux)	Power	Treatment frequency (sessions/week)			Age (years)	SER (D)	AL (mm)	SFCT (μm)		
Xiong et al. (46), 2021	China, Central China	Cohort study, single-center	Ya Kun	/	635	/	Instrument power: 2 ± 0.5 mW	14	74	RLRL	10.22 ± 2.38	−3.39 ± 2.17	25.07 ± 1.15	288.61 ± 56.59	6 m	AL, SER, SFCT
									74	SVL	10.33 ± 2.03	−3.32 ± 1.36	25.07 ± 0.87	286.81 ± 63.67		
Wu et al. (20), 2024	China, North China	Cohort study, single-center	Londa optics	Class 1 laser	635	/	Power: 0.35 mW	14	54	RLRL	8.59 ± 1.51	−1.51 ± 0.97	24.22 ± 0.85	/	6/12/24 m	AL, SER
									56	SVL	8.14 ± 2.10	−1.96 ± 0.88	24.34 ± 0.60	/		
Liu et al. (21), 2024	China, North China	RCT, single-center	Eyerising	/	650 ± 10	1,600	/	10	32	RLRL	9.37 ± 1.69	−2.91 ± 1.27	24.71 ± 0.92	/	6/12 m	AL, SER
									36	SVL	9.55 ± 1.13	−2.61 ± 0.98	24.58 ± 0.64	/		
									40	RLRL	8.95 ± 0.75	0.36 ± 0.32	23.40 ± 0.63	/		
									36	None	8.94 ± 1.09	0.37 ± 0.30	23.30 ± 0.78	/		
Liu et al. (33) 2025	China, North China	RCT, multi-center	Eyerising	/	650 ± 10	1,600	/	10	119	RLRL	10.1 ± 1.7	−7.75 ± 1.91	26.50 ± 1.03	249 ± 69	6/12 m	AL, SER, SFCT
									62	SVL	10.5 ± 1.6	−7.65 ± 1.70	26.38 ± 0.99	261 ± 64		
Chen et al. (34), 2023	China, North China	RCT, single-center	Londa optics	/	635	/	Power: 0.35 mW ± 0.02 mW	14	46	RLRL	9.00 ± 1.90	−2.54 ± 1.04	24.62 ± 0.97	259.00 ± 51.46	6/12 m	AL, SER, SFCT
									40	SVL	8.98 ± 1.92	−2.29 ± 0.77	24.57 ± 0.76	273.08 ± 54.37		
Dong et al. (35), 2023	China, North China	RCT, single-center	Eyerising	/	/	/	RLRL device: 0.29 mW	14	56	RLRL	10.3 ± 2.07	−3.13 ± 1.91	24.7 ± 1.04	/	6 m	AL, SER
							Sham device: 0.03 mW		55	Sham device	9.86 ± 1.41	−2.82 ± 1.86	24.6 ± 0.96	/		
Yang et al. (36), 2025	China, South China	RCT, single-center	LS-03B	/	/	/	Power: 0.39 mW	14	26	RLRL	9.00 (8.00, 10.00)	−1.13 (−1.38, −1.00)	24.30 ± 0.87	/	12 m	AL, SER, SFCT
									26	SVL	9.00 (8.00, 10.00)	−1.13 (−1.25, −1.00)	23.93 ± 0.66			
Tian et al. (37), 2022	China, South China	RCT, single-center	YF020A	Class 1 laser	650	/	/	14	91	RLRL	9.66 ± 1.65	−2.00 (−3.25, −1.25)	24.31 ± 0.92	290.5 (242, 352.5)	6 m	AL, SER, SFCT
									88	SVL	9.47 ± 1.59	−2.00 (−2.75, −1.25)	24.20 ± 0.85	296 (244, 352)		
Tian et al. (38), 2023	China, East China	RCT, single-center	YF020A	Class 1 laser	650	/	/	14	56	RLRL	7.7 ± 1.1	0.25 (−0.25, 0.75)	23.1 ± 0.8	308 (261, 357)	6 m	AL, SER, SFCT
									56	None or SVL	7.9 ± 1.8	0.25 (0.00, 0.75)	23.1 ± 0.7	317 (296, 356)		
Zhou et al. (44), 2022	China, South China	Cohort study, single-center	/	Class 2 laser	635	/	Power: 0.4 mW	14	105	RLRL	9.19 ± 2.40	−3.09 ± 1.74	24.76 ± 1.28	/	6 m	AL, SER
									56	SVL	8.62 ± 2.45	−3.11 ± 1.66	24.75 ± 1.35	/		
Zhu et al. (45), 2024	China, North China	Cohort study, single-center	Eyerising	/	650 ± 10	/	Instrument power: 2 mW	10	53	RLRL	8.96 ± 2.19	−3.02 ± 1.80	24.66 ± 0.93	/	6 m/12 m	AL, SER
							Observation port power: 0.29 mW		55	SVL	8.47 ± 2.10	−2.85 ± 1.71	24.40 ± 1.02	/		
Xiong et al. (17), 2022	China, North China	Cohort study, multi-center	Eyerising	Class 1 laser	650 ± 10	/	/	10	11	RLRL	11.18 ± 1.67	−2.76 ± 1.15	24.58 ± 0.94	/	12 m/24 m	AL, SER, SFCT
									41	SVL	10.79 ± 1.55	−1.77 ± 0.57	24.89 ± 0.94	/		
Xiong et al. (24), 2023	China, East China	RCT, multi-center	/	/	/	/	/	10	60	RLRL	10.52 ± 1.53	−2.30 ± 0.85	24.52 ± 0.68	215.31 ± 58.52	6 m/12 m	AL, SER, SFCT
									60	SVL	10.37 ± 1.61	−2.58 ± 1.15	24.66 ± 0.89	215.50 ± 57.71		
Zhou et al. (39), 2024	China, North China	RCT, single-center	Sky-n1201	Class 1 laser	650	/	Power: 0.37 ± 0.02 mW, 0.60 ± 0.2 mW, 1.2 mW	14	43	RLRL-0.37 mW	8.51 ± 1.51	−1.75 ± 0.96	24.24 ± 0.81	255.09 ± 55.76	6 m	AL, SER, SFCT
									47	RLRL-0.6 mW	8.77 ± 1.43	−2.05 ± 0.88	24.11 ± 0.89	248.11 ± 44.49		
									44	RLRL-1.2 mW	8.68 ± 1.39	−2.10 ± 1.36	24.38 ± 0.90	257.84 ± 63.46		
									43	SVL	8.83 ± 1.53	−2.09 ± 0.90	24.44 ± 0.93	267.58 ± 54.86		
He et al. (40), 2023	China, North China	RCT, single-centre	Eyerising	Class 2a laser	650 ± 10	/	/	10	120	RLRL	8.28 ± 1.10	0.14 ± 0.30	23.36 ± 0.68	/	6/12 m	AL, SER, SFCT
									111	None	8.31 ± 1.07	0.14 ± 0.28	23.30 ± 0.69	/		
Xu et al. (41), 2024	China, Central China	RCT, multi-center	Eyerising	Class 1 laser	650	1,600	Instrument power: 2 mW	14	97	RLRL	10.4 ± 2.4	−5.88 ± 1.69	25.93 ± 1.03	/	6/12 m	AL, SER
							Observation port power: 0.29 mW		95	SVL	11.2 ± 2.1	−5.75 ± 1.17	25.72 ± 0.83	/		
Xiong et al. (22), 2024	China, South China	RCT, single-center	Yishiliang	/	650	700	Instrument power: 0.9 mW	14	36	RLRL	8.83 ± 2.06	−2.47 ± 1.39	24.38 ± 0.87	251.83 ± 65.27	6 m	AL, SER, SFCT
							Observation port power: 0.178 mW		37	SVL	9.00 ± 2.00	−2.22 ± 0.72	24.47 ± 0.58	274.76 ± 63.79		
Jiang et al. (42), 2022	China, East China	RCT, multi-center	Eyerising	Class 1 laser	650 ± 10	1,600	Observation port power: 0.29 mW	10	117	RLRL	10.4 (8.0–13.0)	−2.49 ± 0.92	24.54 ± 0.67	/	6/12 m	AL, SER
									129	SVL	10.5 (8.1–13.0)	−2.67 ± 1.06	24.62 ± 0.86	/		
Jiang et al. (25), 2025	China, East China	RCT, single-centre	Baby Blissful	/	650 ± 10	1,600	/	10	35	RLRL	11.32 ± 2.05	−2.37 ± 0.69	24.59 ± 0.77	244.79 ± 53.11	6/12 m	AL, SER
									35	SVL	11.37 ± 2.08	−2.21 ± 0.71	24.58 ± 0.82	244.94 ± 45.25		
Liu et al. (43), 2023	China, South China	RCT, single-centre	Eyerising	Class 1 laser	650 ± 10	1,600	Observation port power: 0.29 mW	14	43	RLRL	8.98 ± 1.31	0.17 ± 0.35	23.57 ± 0.78	/	6/12 m	AL, SER, SFCT
									42	None	8.95 ± 1.52	0.30 ± 0.35	23.30 ± 0.73	/		

RLRL, repeated low-level red light; RCT, randomized controlled trial; SVLs, single vision lenses; AL, axial length; SER, spherical equivalent refraction; SFCT, subfoveal choroidal thickness.

**Figure 2:**
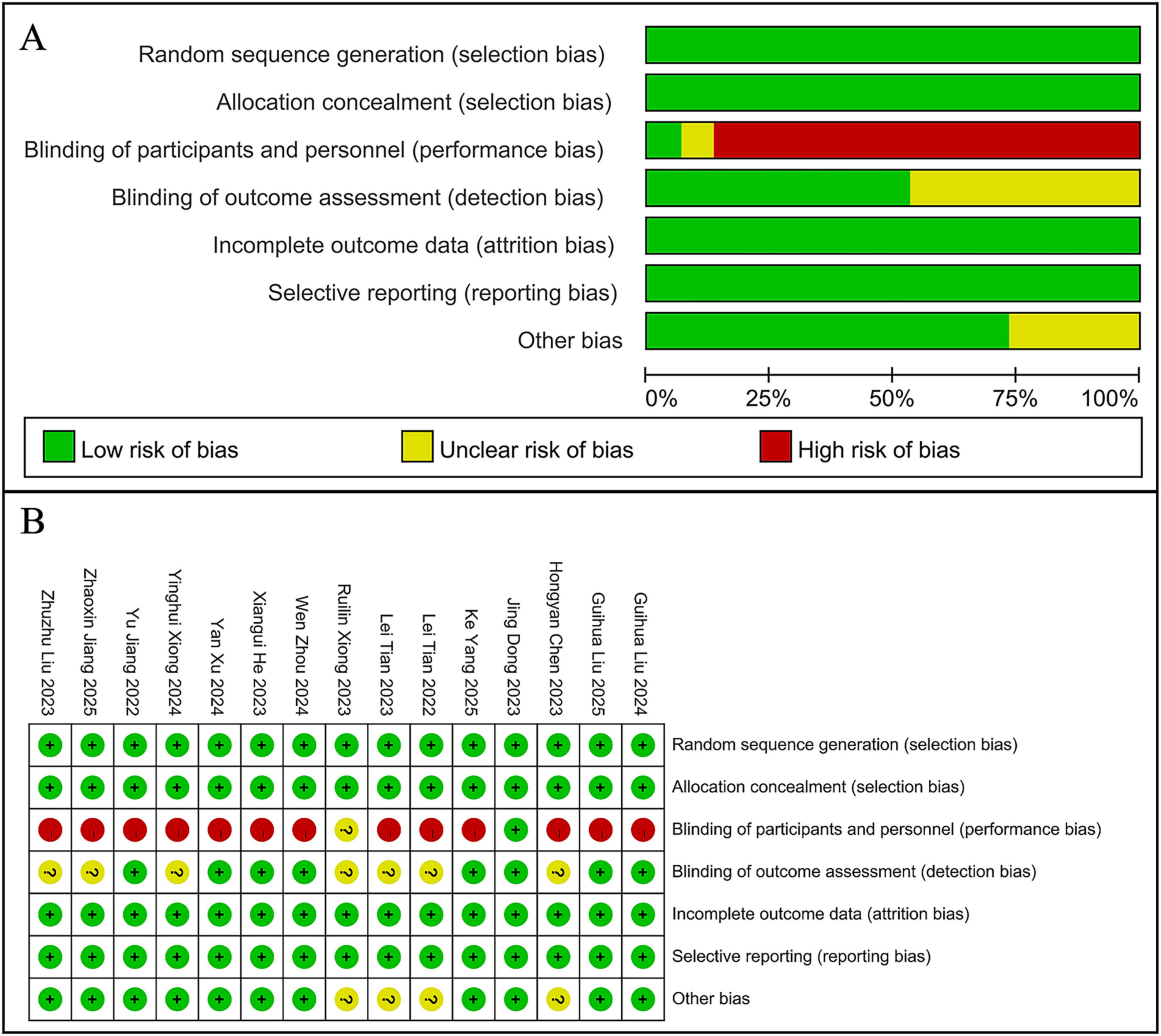
Risk-of-bias assessments of the included studies. **(A)** Risk of bias graph. **(B)** Risk of bias summary.

Only one of the 15 RCTs implemented a randomized, double-blind design, demonstrating a low risk of bias. The remaining RCTs showed varying degrees of bias risk due to methodological differences in study design. The five cohort studies exhibited generally high quality, with scores of at least 7 out of 9 (Figure 2, Table 2).

**Table 2:** Quality assessment of cohort studies using the Newcastle-Ottawa quality assessment scale.

Study	Selection				Comparability of cohorts	Exposure			NOS score
	Exposed cohort representative	Non-exposed cohort selection	Exposure ascertainment	Outcome not present at start		Assessment	Follow-up length	Follow-up adequacy	
Xiong et al. (46)	*	*	*	*	*	*	*	*	8
Wu et al. (20)	*	*	*	*	**	*	*	*	9
Zhou et al. (44)	*	*	*	*	*	*	*		7
Zhu et al. (45)	*	*	*	*	*	*	*	*	8
Xiong et al. (17)	*	*	*	*	*	*	*	*	8

* and ** indicate that the item scores 1 point and 2 points, respectively.

### 3.3 Meta-analysis

#### 3.3.1 Change in AL

**Figure 3:**
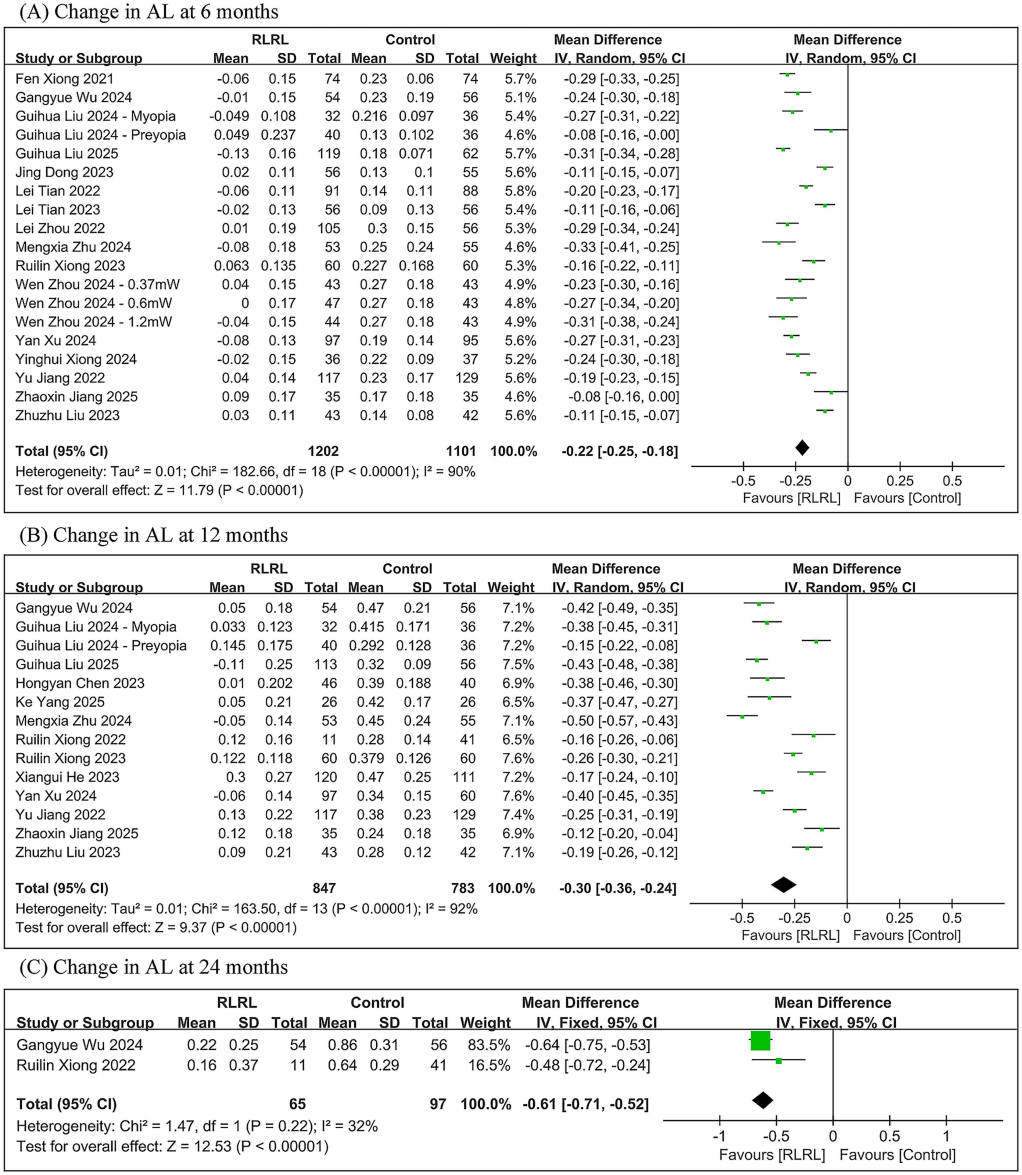
Meta-analysis of the change in AL between the RLRL group and the control group. **(A)** The forest plot of the change in AL at 6 months. **(B)** The forest plot of the change in AL at 12 months. **(C)** The forest plot of the change in AL at 24 months.

The meta-analysis revealed that, compared to controls, the RLRL group showed significantly less axial elongation with between-group differences of −0.22 mm (95% *CI*: −0.25, −0.18; *p* < 0.00001) at 6 months, −0.30 mm (95% *CI*: −0.36, −0.24; *p* < 0.00001) at 12 months, and −0.61 mm (95% *CI*: −0.71, −0.52; *p* < 0.00001) at 24 months, while significant heterogeneity was observed at 6-month (*I*^2^ = 90%) and 12-month (*I*^2^ = 92%) follow-ups but not at 24-month follow-up (*I*^2^ = 32%) (Figure 3).

#### 3.3.2 Change in SER

**Figure 4:**
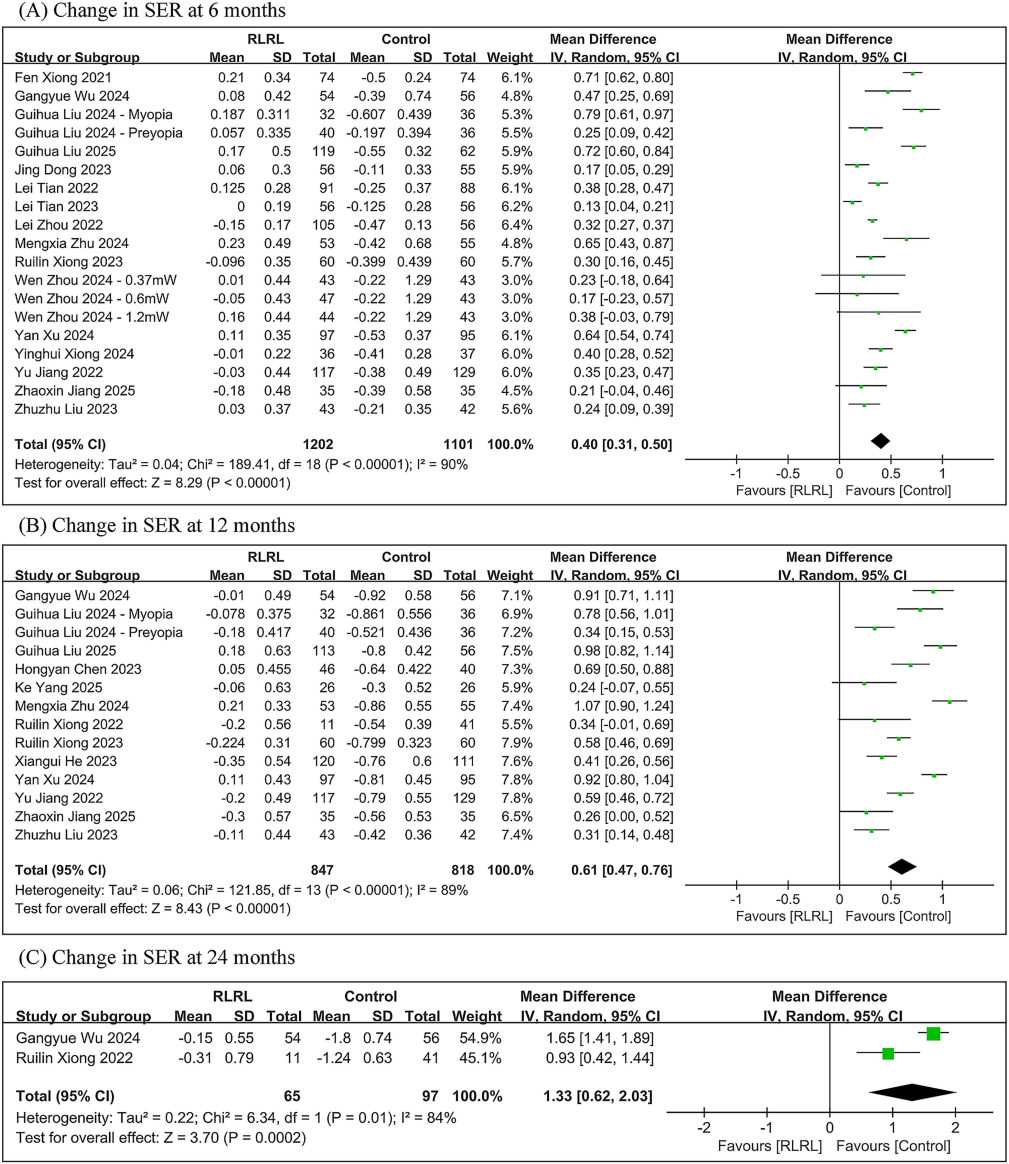
Meta-analysis of the change in SER between the RLRL group and the control group. **(A)** The forest plot of the change in SER at 6 months. **(B)** The forest plot of the change in SER at 12 months. **(C)** The forest plot of the change in SER at 24 months.

The meta-analysis demonstrated that the RLRL group exhibited significantly less change in SER compared to controls, with between-group differences of 0.40 D (95% *CI*: 0.31, 0.50; *p* < 0.00001) at 6 months, 0.61 D (95% *CI*: 0.47, 0.76; *p* < 0.00001) at 12 months, and 1.33 D (95% *CI*: 0.62, 2.03; *p* = 0.0002) at 24 months. Significant heterogeneity was observed across all follow-up periods (6 months: *I*^2^ = 90%; 12 months: *I*^2^ = 89%; 24 months: *I*^2^ = 84%) (Figure 4).

#### 3.3.3 Change in SFCT

**Figure 5:**
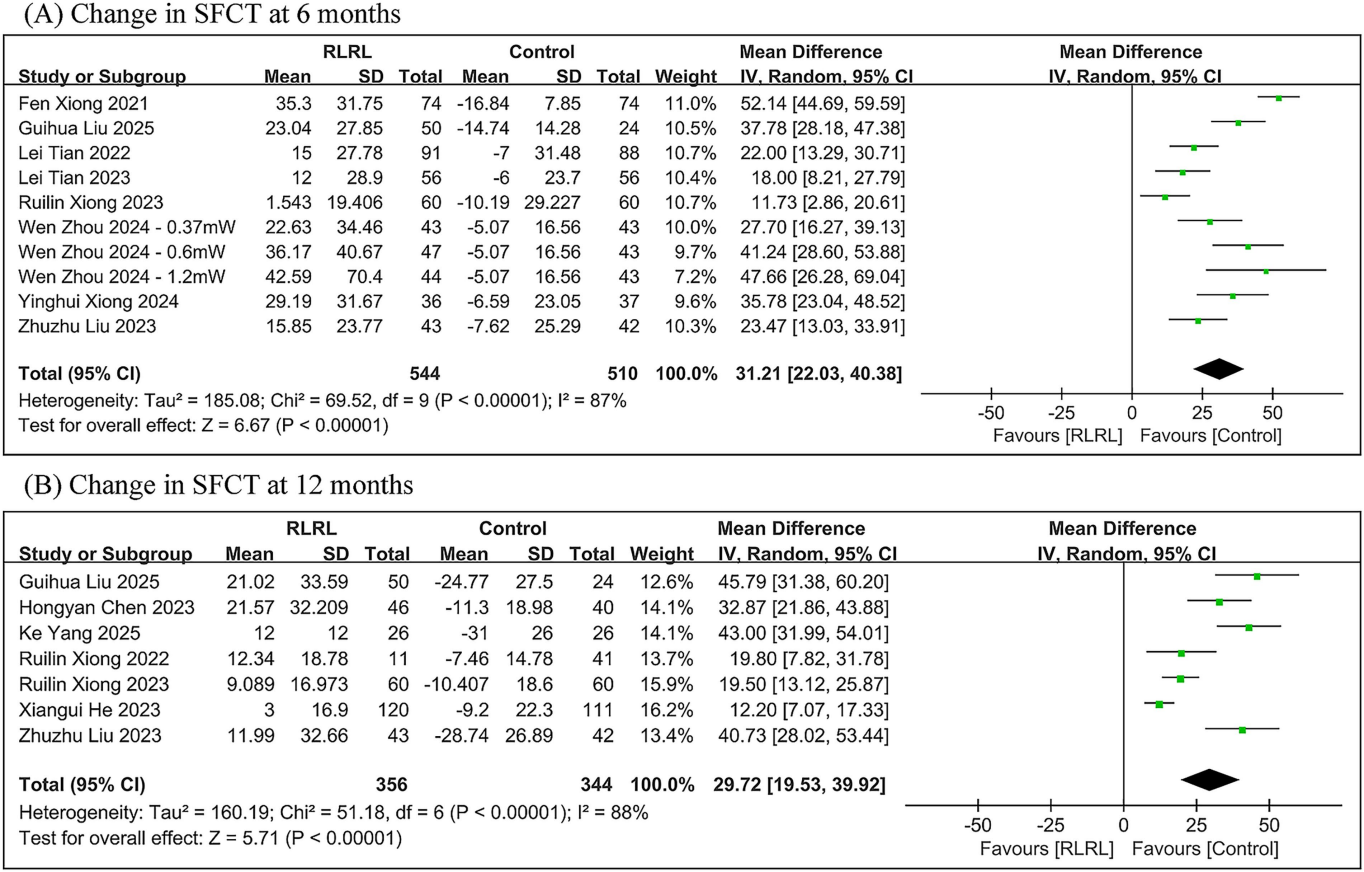
Meta-analysis of the change in SFCT between the RLRL group and the control group. **(A)** The forest plot of the change in SFCT at 6 months. **(B)** The forest plot of the change in SFCT at 12 months.

The meta-analysis demonstrated significantly greater SFCT in the RLRL group compared to the control group, with between-group differences of 31.21 μm (95% *CI*: 22.03, 40.38; *p* < 0.00001) at 6 months and 29.72 μm (95% *CI*: 19.53, 39.92; *p* < 0.00001) at 12 months. Significant heterogeneity was observed throughout all follow-up periods (6 months: *I*^2^ = 87%; 12 months: *I*^2^ = 88%) (Figure 5).

### 3.4 Heterogeneity analysis

This study classifies the included studies according to three methodological approaches: RLRL therapy device (Ya Kun, Londa optics, Eyerising, LS-03B, YF020A, Sky-n1201, Yishiliang, Baby Blissful, unclear), mean of baseline SER (pre-myopia, low myopia, moderate myopia, and high myopia), and treatment frequency (10 sessions/week and 14 sessions/week). A meta-regression analysis demonstrated that the mean of baseline SER constituted the primary source of heterogeneity for all outcome measures at both 6-month and 12-month follow-up periods (all *p* < 0.05; see Table 3 for details). Additionally, the treatment frequency demonstrated a significant correlation with the heterogeneity of the changes in SFCT during both the 6-month (*p* = 0.014) and 12-month (*p* = 0.001) follow-up periods.

**Table 3:** Meta-regression.

Outcome	Covariates	Coefficient	Std. err.	*z*	*p* > |*z*|	[95% conf. interval]
Change in AL at 6 m	RLRL therapy device	0.028	0.043	0.64	0.524	−0.057 to 0.113
	Baseline of SER	−0.562	0.139	−4.04	<0.001	−0.835 to −0.289
	Treatment frequency	0.312	0.21	1.49	0.137	−0.099 to 0.723
	_cons	−0.846	0.441	−1.92	0.055	−1.711 to 0.019
Change in AL at 12 m	RLRL therapy device	0.013	0.088	0.15	0.883	−0.160 to 0.185
	Baseline of SER	−0.515	0.216	−2.39	0.017	−0.939 to −0.092
	Treatment frequency	0.459	0.373	1.23	0.219	−0.273 to 1.191
	_cons	−1.432	0.743	−1.93	0.054	−2.889 to 0.025
Change in SER at 6 m	RLRL therapy device	0.003	0.055	0.05	0.959	−0.104 to 0.110
	Baseline of SER	0.559	0.192	2.92	0.004	0.184 to 0.935
	Treatment frequency	−0.023	0.274	−0.08	0.934	−0.560 to 0.514
	_cons	−0.133	0.55	−0.24	0.809	−1.210 to 0.944
Change in SER at 12 m	RLRL therapy device	−0.041	0.084	−0.49	0.627	−0.204 to 0.123
	Baseline of SER	0.405	0.205	1.98	0.048	0.004 to 0.807
	Treatment frequency	0.004	0.352	0.01	0.990	−0.686 to 0.695
	_cons	0.581	0.703	0.83	0.408	−0.796 to 1.958
Change in SFCT at 6 m	RLRL therapy device	0.035	0.057	0.61	0.541	−0.077 to 0.147
	Baseline of SER	0.577	0.16	3.6	<0.001	0.263 to 0.89
	Treatment frequency	−0.788	0.32	−2.46	0.014	−1.416 to −0.16
	_cons	0.672	0.443	1.52	0.129	−0.195 to 1.54
Change in SFCT at 12 m	RLRL therapy device	0.042	0.038	1.10	0.269	−0.032 to 0.115
	Baseline of SER	0.267	0.096	2.77	0.006	0.078 to 0.456
	Treatment frequency	−0.655	0.193	−3.40	0.001	−1.033 to −0.277
	_cons	1.601	0.350	4.57	0.000	0.914 to 2.287

AL, axial length; SER, spherical equivalent refraction; SFCT, subfoveal choroidal thickness.

Based on the results of the meta-regression analysis, this study divided the subjects into four subgroups (pre-myopia, low myopia, moderate myopia, and high myopia) based on the mean of baseline SER. Furthermore, it subdivided them into six subgroups based on treatment frequency for SFCT during the 6-month and 12-month follow-up periods.

The results of the subgroup analysis demonstrated that, compared with the control group, the therapeutic efficacy of RLRL intensified as the mean of baseline SER deepened. At the 6-month follow-up, RLRL reduced axial elongation by −0.11 mm (95% *CI*: −0.14, −0.08), −0.22 mm (95% *CI*: −0.25, −0.18), −0.28 mm (95% *CI*: −0.31, −0.26), and −0.31 mm (95% *CI*: −0.34, −0.28) and decreased SER progression by 0.18 D(95% *CI*: 0.10, 0.27), 0.39 D (95% *CI*: 0.29, 0.49), 0.55 D (95% *CI*: 0.28, 0.83), and 0.72 D (95% *CI*: 0.60, 0.84) in the pre-myopia group, the low myopia group, the moderate myopia group, and the high myopia group, respectively. At the 12-month follow-up, RLRL reduced axial elongation by −0.17 mm (95% *CI*: −0.21, −0.13), −0.32 mm (95% *CI*: −0.39, −0.24), −0.40 mm (95% *CI*: −0.45, −0.35), and −0.43 mm (95% *CI*: −0.48, −0.38) and decreased SER progression by 0.36 D (95% *CI*: 0.26, 0.46), 0.63 D (95% *CI*: 0.47, 0.79), 0.92 D (95% *CI*: 0.80, 1.04), and 0.98 D (95% *CI*: 0.82, 1.14) in the respective subgroups.

The change in SFCT was influenced by both the mean of baseline SER and the treatment frequency. At the 6-month follow-up, compared with the control group, RLRL therapy increased the SFCT by 20.56 μm (95% *CI*: 13.42, 27.70), 11.73 μm (95% *CI*: 2.86, 20.61), 32.86 μm (95% *CI*: 24.10, 41.62), 52.14 μm (95% *CI*: 44.69, 59.59), and 37.78 μm (95% *CI*: 28.18, 47.38) in the pre-myopia group (14 sessions/week), the low myopia group (10 sessions/week), the low myopia group (14 sessions/week), the moderate myopia group (14 sessions/week), and the high myopia group (10 sessions/week), respectively. At the 12-month follow-up, RLRL therapy increased the SFCT by 12.20 μm (95% *CI*: 7.07, 17.33), 40.73 μm (95% *CI*: 28.02, 53.44), 19.56 μm (95% *CI*: 13.94, 25.19), 37.94 μm (95% *CI*: 28.01, 47.86), and 45.79 μm (95% *CI*: 31.38, 60.20) in the pre-myopia group (10 sessions/week), the pre-myopia group (14 sessions/week), the low myopia group (10 sessions/week), the low myopia group (14 sessions/week), and the high myopia group (10 sessions/week), respectively (Supplementary Figures S1–S3).

### 3.5 Sensitivity analysis

**Figure 6:**
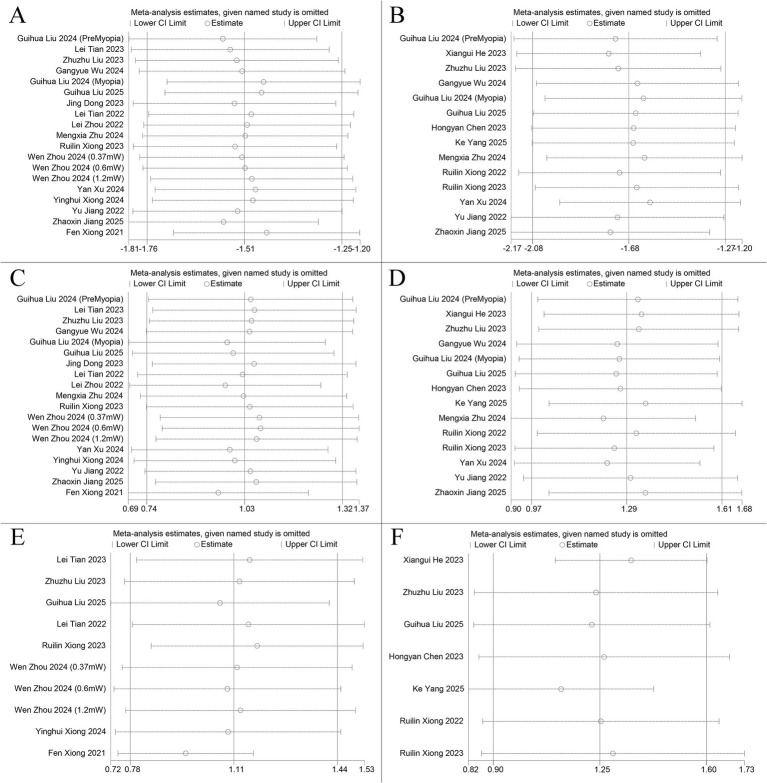
Sensitivity analysis results of each meta-analysis. **(A)** The sensitivity plot of change in AL at 6 months. **(B)** The sensitivity plot of change in AL at 12 months. **(C)** The sensitivity plot of change in SER at 6 months. **(D)** The sensitivity plot of change in SER at 12 months. **(E)** The sensitivity plot of change in SFCT at 6 months. **(F)** The sensitivity plot of change in SFCT at 12 months.

The leave-one-out method was used to evaluate the stability of all meta-analysis outcomes. As shown in Figure 6, no individual study had a significant influence on the results, which remained stable and consistent.

### 3.6 Publication bias

**Figure 7:**
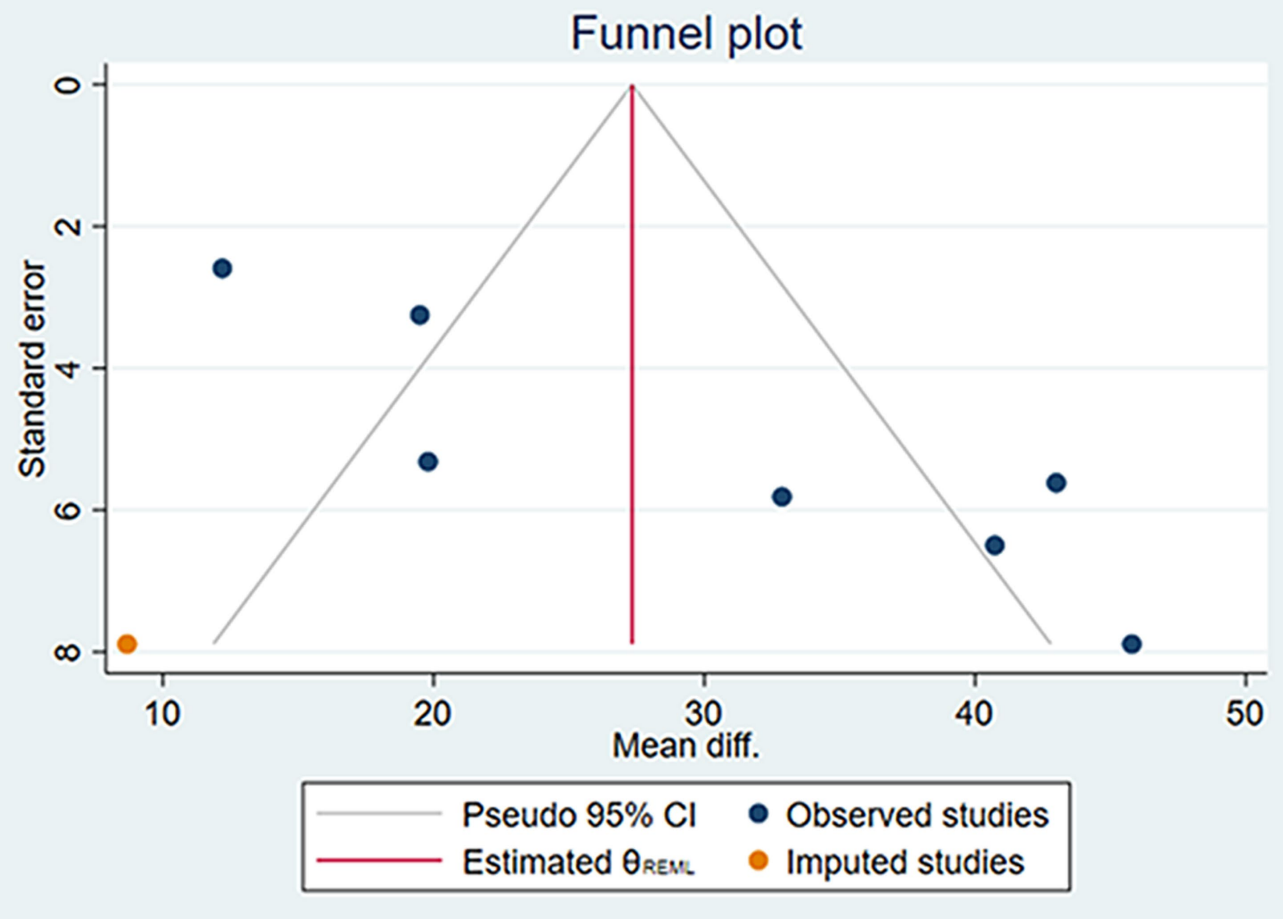
The funnel plot of the changes in SFCT at the 12 months using the non-parametric trim-and-fill method.

The funnel plots for the changes in AL and SER during the 6-month and 12-month follow-up periods, as well as the SFCT at the 6-month follow-up period, were symmetrical (Supplementary Figure S4). Egger’s test revealed no evidence of publication bias in these meta-analyses (all *p* > 0.05; see Table 4). However, the funnel plot for the changes in SFCT at the 12-month follow-up was asymmetrical, and Egger’s test also confirmed the presence of publication bias (*p* = 0.005). The non-parametric trim-and-fill method results suggested that, although publication bias existed in the changes in SFCT at the 12-month follow-up, its influence on the pooled results was relatively minor (Figure 7, Table 5). Additionally, the changes in AL and SER at the 24-month follow-up were not suitable for publication bias assessment due to the inclusion of only two studies.

**Table 4:** Egger’s test.

Outcome	Std_Eff	Coefficient	Std. err.	*t*	*p* > |*t*|	[95% conf. interval]
Change in AL at 6 months	Slope	−1.102	0.710	−1.55	0.139	−2.600 to 0.396
	Bias	−1.843	3.388	−0.54	0.594	−8.991 to 5.306
Change in AL at 12 months	Slope	−0.382	0.636	−0.60	0.559	−1.769 to 1.004
	Bias	−5.452	2.953	−1.85	0.090	−11.887 to 0.982
Change in SER at 6 months	Slope	0.802	0.821	0.98	0.342	−0.930 to 2.534
	Bias	1.092	4.139	0.26	0.795	−7.641 to 9.826
Change in SER at 12 months	Slope	1.067	0.626	1.70	0.114	−0.297 to 2.430
	Bias	1.034	3.025	0.34	0.738	−5.558 to 7.625
Change in SFCT at 6 months	Slope	−0.422	0.918	−0.46	0.658	−2.540 to 1.696
	Bias	7.013	4.344	1.61	0.145	−3.004 to 17.03
Change in SFCT at 12 months	Slope	−0.018	0.239	−0.08	0.943	−0.631 to 0.595
	Bias	5.191	1.086	4.78	0.005	2.399 to 7.984

AL, axial length; SER, spherical equivalent refraction; SFCT, subfoveal choroidal thickness.

**Table 5:** Non-parametric trim-and-fill analysis of publication bias.

Outcome	Studies	Mean diff.	[95% conf. interval]
Change in SFCT at 12 m	Observed	29.542	19.519 to 39.565
	Observed + Imputed	27.333	17.503 to 37.163

SFCT, subfoveal choroidal thickness.

## 4 Discussion

Increasing outdoor activity time is an effective measure for preventing and controlling myopia in children (8, 47, 48). Engaging in at least 11 h of outdoor activities per week with light intensity exceeding 1,000 lux can reduce the risk of rapid myopia progression by 54% (48). Even an additional 40 min of outdoor activity per day can decrease the probability of myopia development in non-myopic children by approximately 23% over the next 3 years (8). Meta-analyses further confirm that increasing outdoor activity time not only effectively prevents the onset of myopia in non-myopic children but also slows the progression of myopia in those who are already myopic (49).

The role of this light environment regulation in myopia prevention and control could be achieved through biological mechanisms such as the retinal dopaminergic system and hemodynamic changes. Animal experimental studies (50–52) have shown that light, as a key environmental factor affecting myopia progression, has a mechanism of action that is not only related to intensity and duration but also closely tied to the biological effects of specific light wavelengths. In myopia models of rhesus monkeys and tree shrews, narrowband long-wave light has been shown to significantly inhibit the increase in AL caused by form deprivation or hyperopic defocus (14, 53). This protective effect may be related to the photoregulation mechanism of the retinal dopaminergic system, where different wavelengths of light can differentially regulate dopamine secretion and metabolism (54). As a key neurotransmitter in eye growth regulation (55), dopamine not only directly participates in the emmetropization process but also promotes choroidal thickening by regulating the release of other transmitters, such as nitric oxide, thereby counteracting abnormal axial elongation (56–58). Oxidative stress and inflammatory responses may contribute to the pathological process of myopia by disrupting the aforementioned neurotransmitter regulation pathways (59). Especially under hypoxic conditions, oxidative damage can significantly impair the normal regulatory function of the nitric oxide and dopamine systems (60, 61).

From a hemodynamic perspective, dopamine can exert a protective effect by enhancing retinal perfusion and choroidal blood flow (62). Given that the choroid has dual functions, providing nutrition to the retina and regulating refraction (63, 64), the reduction in its blood flow is considered an important inducer of choroidal and retinal thinning and axial elongation during the development of myopia (65). This hypothesis is supported by clinical observations, which often reveal reduced ocular blood flow in myopic patients (66–68). In contrast, researchers have observed that low-concentration atropine (69), defocus incorporated multiple-segment spectacle lenses (70), and orthokeratology (71, 72) can induce choroidal thickening and changes in blood flow in the intervention of childhood myopia progression. Although the causal relationship between reduced blood flow and tissue thinning remains controversial (2), animal experiments have confirmed that narrowband long-wavelength light can induce vitreous cavity depth reduction and refractive changes related to choroidal thickening in tree shrews (73, 74). These studies provide a theoretical basis for RLRL intervention in the progression of childhood myopia. Some researchers (34, 42) have proposed a hypothesis that RLRL may decrease scleral hypoxia by increasing choroidal blood flow, reducing oxidative stress and inflammation, thereby controlling myopia progression (75–77).

Multiple RLRL clinical studies (33, 34, 37, 39, 44) have shown that it can induce choroidal thickening and slow axial elongation in myopic children. On the other hand, in terms of meta-analyses, Youssef et al. (26) verified the effectiveness of RLRL treatment in delaying childhood myopia. However, the meta-analysis was unable to further investigate the sources of heterogeneity in the studies, and the stability of the results and potential publication bias require further verification due to the limited number of studies included (a total of 5 studies). Similar problems also exist in previously published related meta-analyses (each meta-analysis included less than 10 studies), which, to some extent, weaken the statistical power of these meta-analyses (27, 28, 78).

This study evaluated the effect of RLRL treatment on the prevention and control of childhood myopia through a meta-analysis. The results showed that, compared with the control group, the RLRL group had a significant advantage in delaying axial elongation, with *MDs* of −0.21 mm, −0.30 mm, and −0.61 mm at 6, 12, and 24 months of follow-up, respectively; the *MDs* of SE changes were 0.39 D, 0.61 D, and 1.33 D, respectively; the *MDs* of SFCT increased by 28.12 μm and 29.72 μm at 6 and 12 months of follow-up, respectively. The results indicate that RLRL intervention can effectively delay the progression of myopia in children, and this effect may be related to the SFCT thickening induced by RLRL (65). It is worth noting that, given the heterogeneity in the current RLRL meta-analysis results, this study, benefiting from the large number of included studies (a total of 20), employed meta-regression and subgroup analysis for the first time to explore the possible sources of heterogeneity. The results showed that the mean of baseline SER and the treatment frequency were the primary sources of heterogeneity in this study. Children with more severe baseline myopia may achieve better AL and SER control effects through RLRL; higher treatment frequency may also induce thicker SFCT changes in myopic children. This conclusion is similar to the findings of Liu et al. (21), whose study showed that RLRL was more effective in myopic children than in pre-myopic children.

Notably, in the Low Myopia subgroup, there was still significant heterogeneity in AL, SE, and SFCT at 6 months of follow-up and in AL and SE at 12 months of follow-up, suggesting that this phenomenon may be related to the subgroup grouping strategy. Although the association between baseline SER and treatment efficacy has been established in the meta-regression, the subgroup grouping method based on the mean of baseline SER can only partially explain the sources of heterogeneity. We speculate that this phenomenon may be related to the lack of sample stratification based on baseline SER in the original studies. The differences in sample characteristics among the original studies may be a potential source of residual heterogeneity in the Low Myopia subgroup.

Based on the analysis of existing literature, this study first reported the presence of publication bias in RLRL treatment studies affecting SFCT. This bias was confirmed by the asymmetric funnel plot of the SFCT indicator and Egger’s test (*p* = 0.005). Despite the presence of publication bias, the non-parametric trim-and-fill analysis revealed that its impact on the results was minimal. Before and after the trim-and-fill analysis, the changes in SFCT at 12 months of follow-up were 29.542 μm (95% *CI*: 19.519, 39.565) and 27.333 μm (95% *CI*: 17.503, 37.163), respectively. Combined with the results of the sensitivity analysis, these findings indicate that the results of this study have good stability.

Furthermore, although this study has confirmed the efficacy of RLRL in myopia intervention, several aspects of the current RLRL therapy remain to be further explored, including the dose–response relationship, the myopia rebound effect, and treatment safety. First of all, regarding the dose–response relationship, Jiang et al.’s study (42) demonstrated that, in the RLRL group, as treatment compliance increased from <50 to >75%, the control effects on AL and SER increased from 44.6 and 41.7% to 76.8 and 87.7%, respectively. Dong et al. (35) found that an RLRL device with a power of 0.29 mW had better myopia control effects than an RLRL treatment device with a power of 0.03 mW. Zhou et al.’s study (39) did not reveal differences in the efficacy of three RLRL powers (0.37 mW, 0.60 mW, and 1.20 mW) on controlling childhood myopia. However, the trend indicated that higher power might have better efficacy. Our results also showed that SFCT was affected by the frequency of treatment. These research results collectively demonstrate the potential dose–effect relationship of RLRL treatment.

Second, RLRL therapy may induce a myopia rebound effect, wherein the progression of myopia accelerates significantly after the discontinuation of intervention compared to the treatment period. Chen et al. (34) first reported a mild rebound effect following RLRL therapy, showing that, during treatment, AL and SER increased by 0.01 mm and 0.05 D, respectively, whereas, within 3 months post-treatment, AL and SER increased by 0.16 mm and −0.20 D, respectively. Xiong et al. (17) further confirmed this phenomenon, revealing that the RLRL-single vision spectacle (SVS) group exhibited greater AL and SER increases (0.42 mm and −0.91 D, respectively) in the second year after treatment cessation compared to the SVS-SVS group (0.28 mm and −0.54 D, respectively). Notably, this rebound effect is not unique to RLRL therapy, as other myopia interventions, such as atropine (79, 80) and orthokeratology (81), also demonstrate similar rebound characteristics.

Finally, concerning treatment safety, early studies (34, 37, 40, 41, 45) primarily relied on questionnaires to assess subjective symptoms and employed fundus photography, ultrasonography, and optical coherence tomography to detect retinal structural damage. Multiple systematic reviews (28, 78, 82) have shown no evidence of irreversible structural changes or vision loss in the short term. However, existing evidence remains insufficient to confirm the long-term safety of RLRL fully. Ostrin et al. (83) believe that current adverse event metrics lack sensitivity to subtle light-induced damage. Even exposure within international safety thresholds may induce subclinical damage to the retinal pigment epithelium and photoreceptor layers (84–86). A recent high-resolution adaptive optics scanning laser ophthalmoscopy study (84) further revealed that myopic children receiving RLRL therapy for at least 1 year exhibited reduced parafoveal cone density and a 7.23-fold increased risk of abnormal signals. Therefore, a comprehensive assessment of the long-term safety of RLRL therapy remains an indispensable component of future research.

This study has the following limitations: (1) The included literature is limited to studies published in Chinese and English, and the subjects are all Chinese children, which may introduce language bias and limit the generalizability of the research results to other regions and ethnic groups; (2) due to the specificity of the intervention measures, except for the study by Dong et al. (35), which used a sham device group, the remaining RCTs did not implement a double-blind design; (3) in terms of follow-up time, most studies had a follow-up period of 6 or 12 months, and only two studies (17, 20) completed a 24-month follow-up. Therefore, it is necessary to further evaluate the long-term effects of RLRL therapy on children’s AL, SER, and SFCT; (4) due to the lack of stratified analysis based on baseline characteristics in the original studies, there may be potential residual heterogeneity in subgroup analysis, indicating the need for further evaluation of the efficacy differences of RLRL in children with different degrees of myopia; and (5) although meta-regression clarified the sources of heterogeneity, the number of SFCT-related studies in subgroup analysis was limited, which may have weakened the statistical power. Therefore, it is recommended to expand the sample size in the future to improve the reliability of the conclusions.

## 5 Conclusion

In summary, this meta-analysis demonstrates that RLRL treatment has significant clinical effects on pre-myopic and myopic Chinese children, including increasing SFCT thickness, delaying AL elongation, and reducing SER progression. The treatment effect is related to the degree of myopia in children. However, existing studies lack long-term safety assessments, and their treatment effects may be influenced by dose-effect relationships, carrying the risk of myopia rebound after discontinuation of the intervention. Therefore, future research should conduct larger-scale, longer-term clinical studies, especially stratified studies targeting children with varying degrees of myopia, to determine the optimal prevention and control effect of RLRL, while ensuring safety through more rigorous experimental designs.

## Data Availability

The original contributions presented in the study are included in the article/Supplementary material, further inquiries can be directed to the corresponding authors.
