# Supplementary figures and images for: Effects of repeated low-level red light therapy on myopia progression in children: a systematic review and meta-analysis
Source: Front Med (Lausanne). 2025 Aug 13;12:1640403. doi: 10.3389/fmed.2025.1640403 (PMC12380900; doi:10.3389/fmed.2025.1640403)

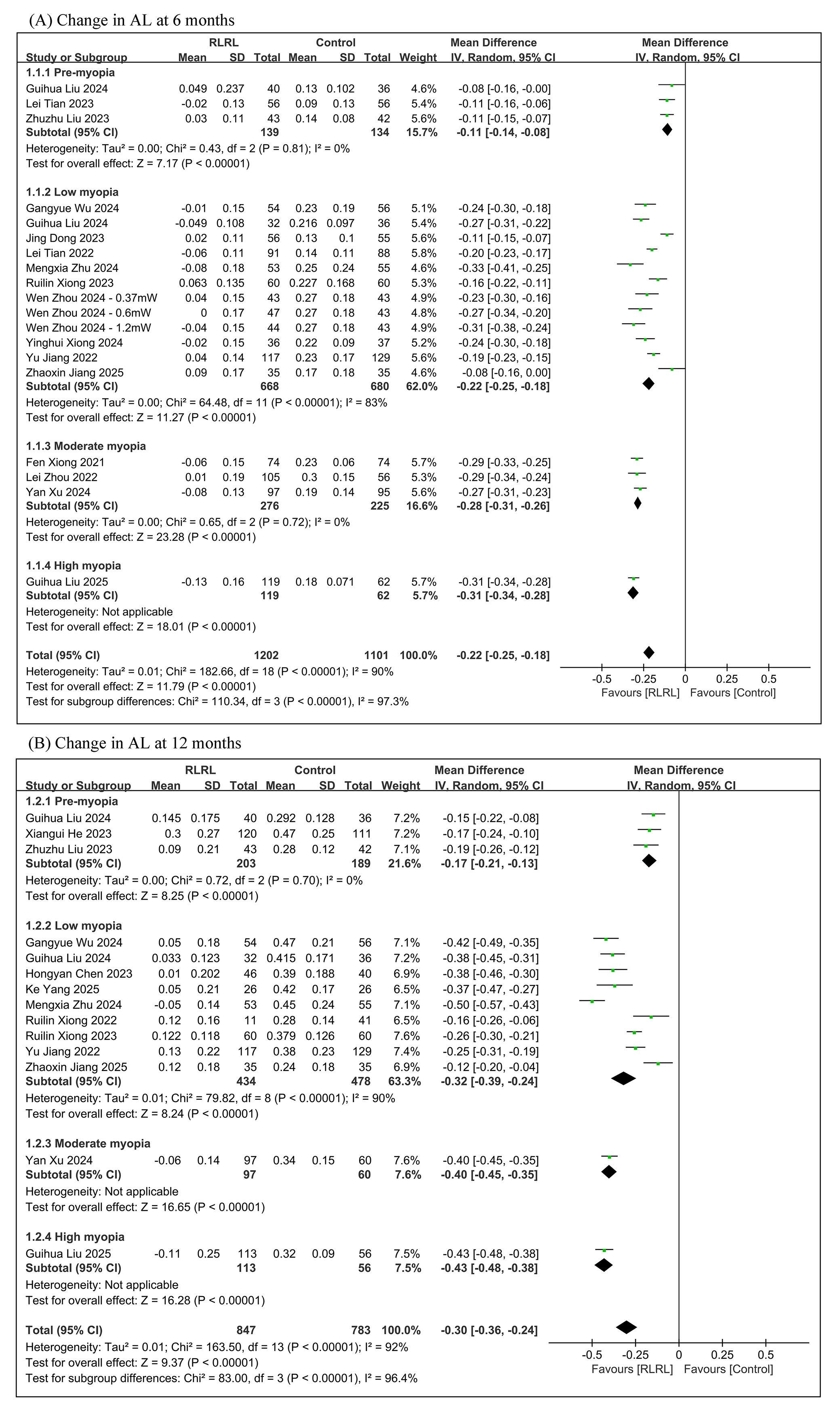

Supplement: Supplementary file 1 [file Image_1.tif]

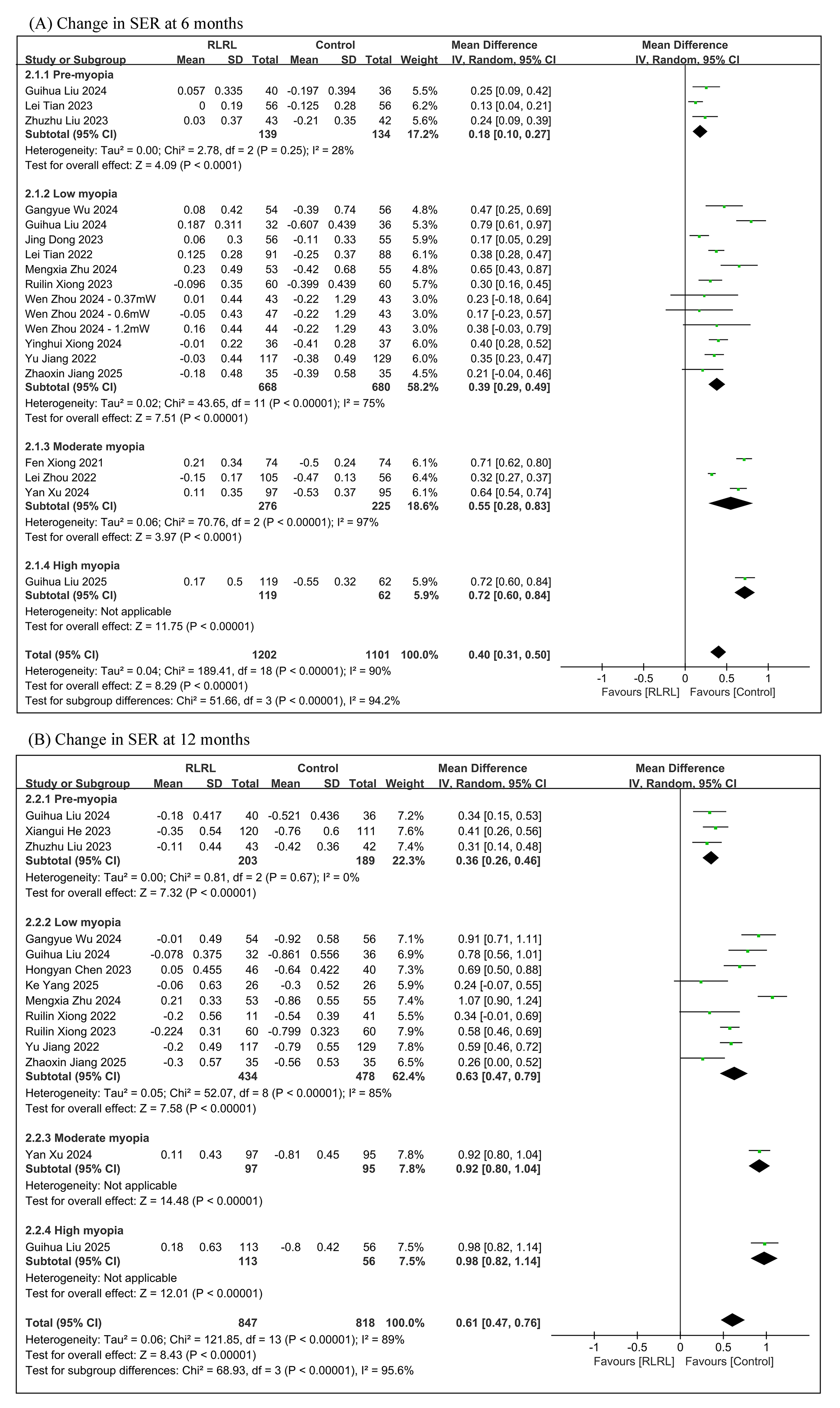

Supplement: Supplementary file 2 [file Image_2.tif]

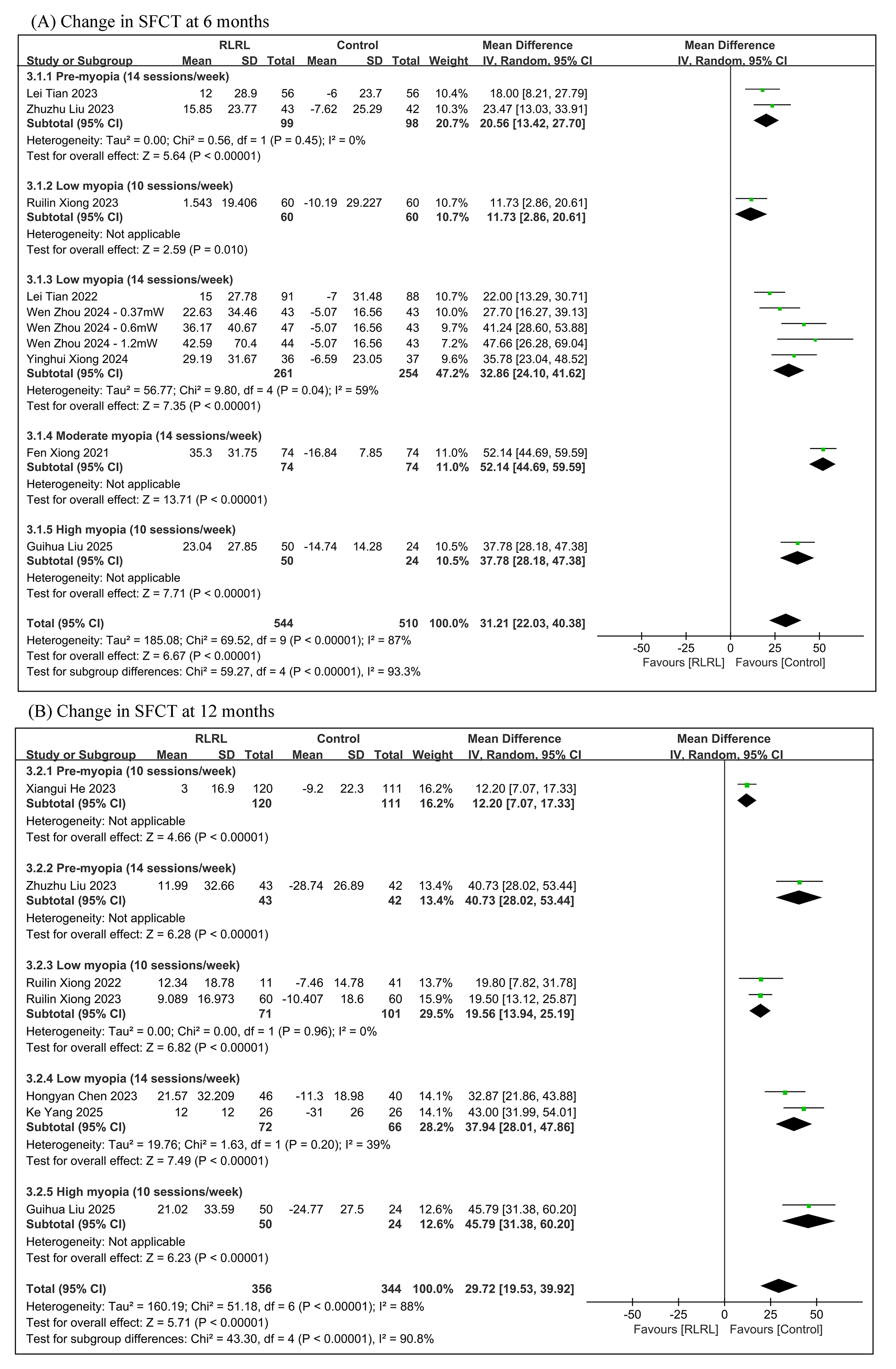

Supplement: Supplementary file 3 [file Image_3.tif]

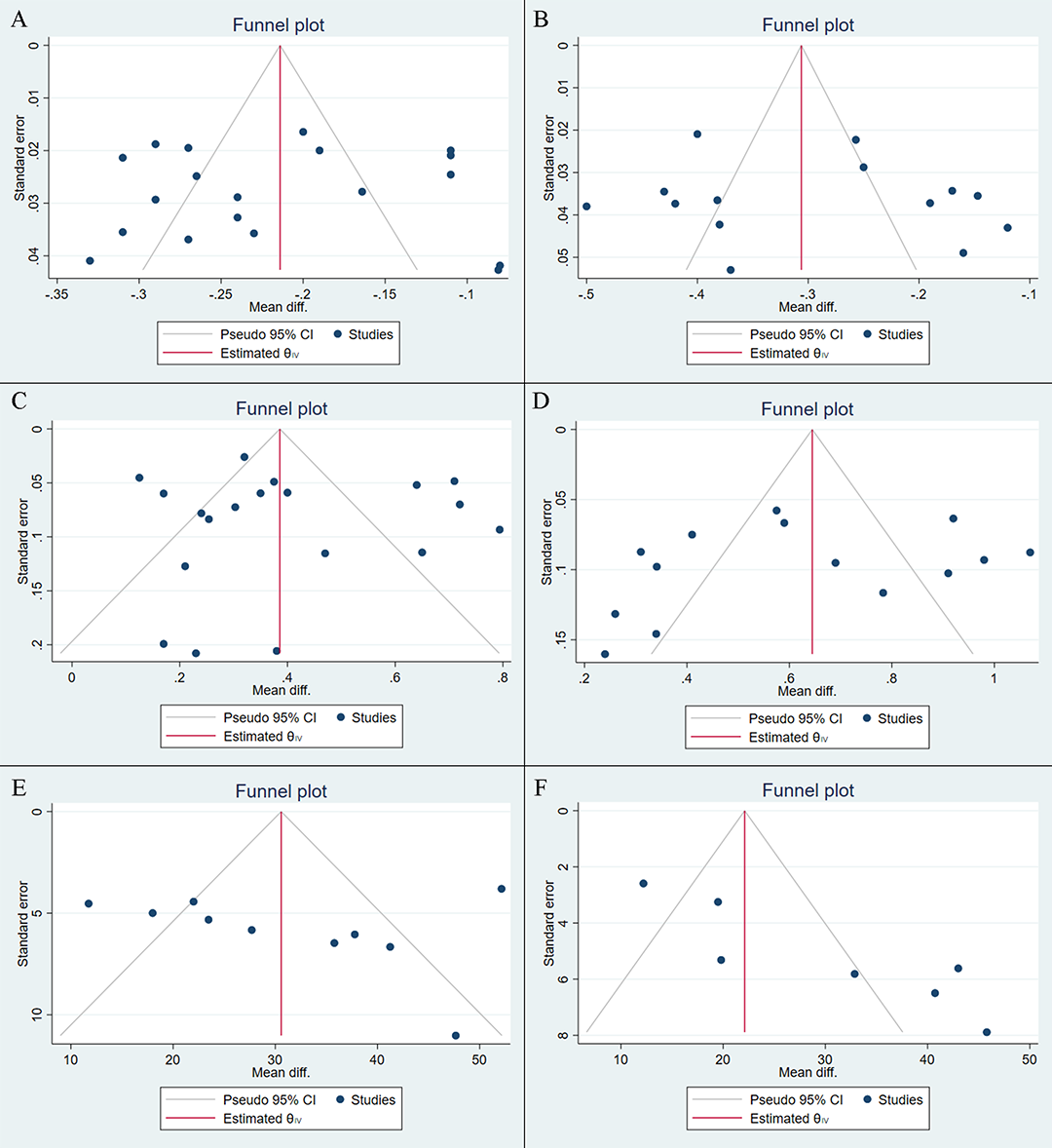

Supplement: Supplementary file 4 [file Image_4.tif]
