# Supplementary material for: Effects of repeated low-level red light therapy on myopia progression in children: a systematic review and meta-analysis
Source: Front Med (Lausanne). 2025 Aug 13;12:1640403. doi: 10.3389/fmed.2025.1640403 (PMC12380900; doi:10.3389/fmed.2025.1640403)
Supplement: Supplementary file 6 [file Table_2.docx]

**Supplementary file 2. Search strategies modified**

**Appendix.**

**Search strategies modified in PubMed (a), the Cochrane Library (b), Embase (c), Web of Science (d) and CNKI(e).**

**a. Search strategy in PubMed**

| # | Search syntax | Citations found |
| --- | --- | --- |
| 1 | "myopia"[MeSH Terms] OR "myopia"[All Fields] OR "myopias"[All Fields] |  |
| 2 | "Red light"[MeSH Terms] OR "Photonic stimulation"[MeSH Terms] OR "Photobiomodulation"[MeSH Terms] OR "Phototherapy"[MeSH Terms] OR "Lowpower laser therapy"[MeSH Terms] |  |
| 3 | "child"[MeSH Terms] OR "child"[All Fields] OR "children"[All Fields] OR "child's"[All Fields] OR "children's"[All Fields] OR "childrens"[All Fields] OR "childs"[All Fields] |  |
| 4 | #1 AND #2 AND #3 | 112 |

**b. Search strategy in the Cochrane Library**

| # | Search syntax | Citations found |
| --- | --- | --- |
| 1 | (myopia):ti,ab,kw OR (nearsightedness):ti,ab,kw OR (refractive errors):ti,ab,kw OR (short-sightedness):ti,ab,kw |  |
| 2 | (low-level red-light therapy):ti,ab,kw OR (LLRL):ti,ab,kw OR (low-level laser light therapy):ti,ab,kw OR (lowpower laser therapy):ti,ab,kw AND (biostimulation):ti,ab,kw |  |
| 3 | (photonic stimulation):ti,ab,kw OR (photobiomodulation):ti,ab,kw OR (phototherapy):ti,ab,kw OR (red light therapy):ti,ab,kw AND (low-level red light):ti,ab,kw |  |
| 4 | (Repeated Low-Level Red Light):ti,ab,kw OR (RLRL):ti,ab,kw |  |
| 5 | #2 OR #3 OR #4 |  |
| 6 | children |  |
| 7 | #1 AND #5 AND #6 | 44 |

**c. Search strategy in Embase**

| # | Search syntax | Citations found |
| --- | --- | --- |
| 1 | myopia |  |
| 2 | nearsightedness |  |
| 3 | refractive errors |  |
| 4 | short-sightedness |  |
| 5 | #1 OR #2 OR #3 OR #4 |  |
| 6 | low-level red-light therapy |  |
| 7 | LLRL |  |
| 8 | low-level laser light therapy |  |
| 9 | lowpower laser therapy |  |
| 10 | biostimulation |  |
| 11 | photonic stimulation |  |
| 12 | photobiomodulation |  |
| 13 | phototherapy |  |
| 14 | red light therapy |  |
| 15 | low-level red light |  |
| 16 | Repeated Low-Level Red Light |  |
| 17 | RLRL |  |
| 18 | #6 OR #7 OR #8 OR #9 OR #10 OR #11 OR #12 OR #13 OR #14 OR #15 OR #16 OR #17 |  |
| 19 | children |  |
| 20 | #5 AND #18 AND #19 | 107 |

**d. Search strategy in Web of Science**

| # | Search syntax | Citations found |
| --- | --- | --- |
| 1 | myopia |  |
| 2 | nearsightedness |  |
| 3 | refractive errors |  |
| 4 | short-sightedness |  |
| 5 | #1 OR #2 OR #3 OR #4 |  |
| 6 | low-level red-light therapy |  |
| 7 | LLRL |  |
| 8 | low-level laser light therapy |  |
| 9 | lowpower laser therapy |  |
| 10 | biostimulation |  |
| 11 | photonic stimulation |  |
| 12 | photobiomodulation |  |
| 13 | phototherapy |  |
| 14 | red light therapy |  |
| 15 | low-level red light |  |
| 16 | Repeated Low-Level Red Light |  |
| 17 | RLRL |  |
| 18 | #6 OR #7 OR #8 OR #9 OR #10 OR #11 OR #12 OR #13 OR #14 OR #15 OR #16 OR #17 |  |
| 19 | children |  |
| 20 | #5 AND #18 AND #19 | 66 |

**e. Search strategy in CNKI**

| # | Search syntax | Citations found |
| --- | --- | --- |
| 1 | 近视 |  |
| 2 | 屈光不正 |  |
| 3 | #1 OR #2 |  |
| 4 | 红光 |  |
| 5 | 红光治疗 |  |
| 6 | 红光疗法 |  |
| 7 | 重复低强度红光 |  |
| 8 | #4 OR #5 OR #6 OR #7 |  |
| 9 | 儿童 |  |
| 10 | 青少年 |  |
| 11 | #8 OR #9 |  |
| 12 | #3 AND #8 AND #11 | 58 |
